# Comparative Effectiveness of Remdesivir in Hospitalized COVID-19 Patients: A Retrospective Cohort Study From the Pre-vaccination and Omicron Eras

**DOI:** 10.7759/cureus.87561

**Published:** 2025-07-08

**Authors:** Josef Yayan

**Affiliations:** 1 Internal Medicine, Pneumology, Witten/Herdecke University, Helios University Hospital Wuppertal, Wuppertal, DEU

**Keywords:** antiviral therapy, comparative study, covid-19, hospitalization, mortality, omicron variant, real-world evidence, remdesivir

## Abstract

Background: The real-world effectiveness of remdesivir for COVID-19 pneumonia remains a subject of debate, particularly across different phases of the pandemic. This study compares clinical outcomes and inflammatory biomarker profiles in hospitalized patients treated with remdesivir during two distinct periods: the pre-vaccination era in 2020 and the Omicron-dominant, post-vaccination period in 2023.

Methods: This retrospective observational study was conducted at a tertiary care hospital in Germany. Adults hospitalized with polymerase chain reaction (PCR)-confirmed COVID-19 pneumonia were included from two timeframes: 2020 (n = 154) and 2023 (n = 178). Patients were stratified by remdesivir treatment. The primary outcome was length of hospital stay; secondary outcomes included ICU admission, mechanical ventilation, in-hospital mortality, oxygen requirement, WHO COVID-19 severity grade, and admission inflammatory markers.

Results: In 2020, 38 patients (24.7%) received remdesivir; in 2023, 64 patients (36.0%) were treated with the drug. Remdesivir use was associated with significantly shorter hospital stays among survivors in both periods (2020: median 10 vs. 20 days; 2023: median 7 vs. 13 days; both p < 0.01). Most treated patients required oxygen and met WHO clinical severity grades 4-6. Inflammatory markers at admission were lower in survivors and the 2023 cohort. In-hospital mortality was 16.9% in 2020 and 9.0% in 2023. A trend toward lower mortality was observed in 2023, although not statistically significant.

Conclusion: Remdesivir use was consistently associated with reduced hospital stays across both pandemic phases. A potential mortality benefit emerged during the Omicron era, particularly in high-risk patients requiring oxygen, supporting the continued clinical use of remdesivir during periods of high transmission.

## Introduction

Remdesivir, an antiviral initially developed for Ebola, has played a central role in the treatment of COVID-19 since early 2020. Early real-world and clinical trial data demonstrated promising outcomes. For instance, Mozaffari et al. reported improved survival among immunocompromised patients across different SARS-CoV-2 variant waves [1]. Spinner et al. confirmed benefits in patients with moderate disease [2], and Beigel et al. observed improved recovery in severe cases [3].

Systematic reviews and meta-analyses have supported the effectiveness of remdesivir, particularly in hospitalized and elderly populations [4,5]. Clinical outcomes, however, are influenced by biological variables such as sex, age, and comorbidities. Male sex has been associated with increased mortality risk [6], and biomarkers such as lactate dehydrogenase (LDH) and C-reactive protein (CRP) have been predictive of adverse outcomes [7]. These inflammatory markers are often elevated in patients with multiple comorbidities, a known risk factor for poor prognosis [8].

Other routinely measured laboratory parameters, including white blood cell (WBC) count and lymphocyte percentage, also hold prognostic value and are commonly used in clinical risk stratification [9]. With the emergence of the Omicron variant, updated real-world studies by Mozaffari et al. [10] and Kuritzkes [11] confirmed that remdesivir remains effective in high-risk patients, including those with immunosuppression and advanced age. Baseline clinical and biomarker characteristics, originally described in early pandemic cohorts by Guan et al. [12] and Huang et al. [13], continue to guide treatment decisions.

The present retrospective cohort study compares clinical outcomes and inflammatory biomarker profiles among hospitalized COVID-19 patients treated with remdesivir across two distinct pandemic phases: the pre-vaccination era (2020) and the Omicron-dominant, post-vaccination era (2023). In addition, this study aims to identify patterns in hospital length of stay, mortality, oxygen therapy, and WHO clinical severity classification to better inform the continued use of remdesivir in current practice.

## Materials and methods

Study design and setting

This retrospective observational study was conducted at the Clinic for Internal Medicine, Lung Diseases and Infectiology, Lüdenscheid Hospital, Germany, a tertiary care center. The study included adult patients (≥18 years) hospitalized with polymerase chain reaction (PCR)-confirmed COVID-19 pneumonia during two distinct time periods: January to December 2020 (pre-vaccination era) and January to June 2023 (Omicron/post-vaccination era).

Inclusion and exclusion criteria

Eligible patients had radiologically confirmed pneumonia and a positive SARS-CoV-2 PCR test. Exclusion criteria included incomplete clinical records, transfer from external hospitals after the acute phase, and initiation of antiviral therapy more than 10 days after symptom onset.

Patient stratification

Patients were grouped based on whether they received remdesivir during hospitalization. The decision to administer remdesivir followed national guidelines and physician judgment and was generally based on oxygen requirement, symptom duration of less than 10 days, and preserved renal and hepatic function. Patients receiving remdesivir typically required supplemental oxygen and corresponded to WHO COVID-19 Clinical Progression Scale grades 4-6.

Data collection

Demographic data, comorbidities, symptoms at admission, laboratory markers (C-reactive protein (CRP), lactate dehydrogenase (LDH), procalcitonin (PCT), leukocyte and lymphocyte counts), imaging findings, ICU admission, mechanical ventilation, remdesivir administration, and clinical outcomes (length of stay and in-hospital mortality) were extracted from the hospital information system. All inflammatory and biochemical markers were collected on the day of hospital admission, prior to or at the start of remdesivir therapy, in order to reflect early disease severity.

Outcomes

The primary outcome was hospital length of stay among survivors. Secondary outcomes included in-hospital mortality, ICU admission, mechanical ventilation, oxygen requirement, WHO COVID-19 severity grade, and inflammatory marker profiles at admission.

Statistical analysis

Continuous variables are presented as means with standard deviations or medians with interquartile ranges, depending on distribution. Categorical variables are shown as counts and percentages (n, %). Group comparisons were performed using Student’s t-test or Mann-Whitney U test for continuous variables and chi-square test or Fisher’s exact test for categorical variables. Test statistics, including degrees of freedom (df) and effect size (phi coefficient, φ), are reported for chi-square tests. A two-sided p value < 0.05 was considered statistically significant. Analyses were conducted using VassarStats.

Ethics statement

The study was conducted in accordance with the Declaration of Helsinki and approved by the Ethics Committee of Witten/Herdecke University (approval code: S-123/2023, approval date: 26 May 2023).

Informed consent statement

Patient consent was waived due to the retrospective and anonymized nature of the study.

## Results

Hospitalized patient overview

A total of 332 patients with PCR-confirmed COVID-19 pneumonia were included in the analysis, with 154 patients hospitalized in 2020 and 178 patients in 2023 (Table 1). The mean age of the total cohort was 63.4 ± 15.8 years; 188 (56.6%) were male (2020: n = 83, 53.9%; 2023: n = 105, 59.0%).

**Table 1:** Patient characteristics and outcomes by year

Variable	2020 year	2023 year	Chi-square / p / φ
Age (mean ± SD)	59.5 ± 15.9	61.7 ± 16.1	t-test, p = 0.82
Male gender	n = 83 (53.9%)	n = 105 (59.0%)	χ²(1) = 0.86, p = 0.35, φ = 0.05
Remdesivir use	n = 38 (24.7%)	n = 64 (36.0%)	χ²(1) = 4.26, p = 0.039, φ = 0.12
Mortality	n = 26 (16.9%)	n = 16 (9.0%)	χ²(1) = 3.92, p = 0.048, φ = 0.11

Remdesivir use and demographics

In 2020, 38 of 154 patients (24.7%) received remdesivir, compared to 64 of 178 patients (36.0%) in 2023. Patients treated with remdesivir in 2020 were significantly older than those not receiving it (mean age: 71.1 ± 10.9 vs. 59.5 ± 15.9 years; p = 0.0011). In 2023, the age difference between groups was not significant (remdesivir: 62.1 ± 14.4 years vs. no remdesivir: 61.7 ± 16.1 years; p = 0.82) (Table 2).

**Table 2:** Clinical outcomes among survivors by remdesivir treatment

Variable	2020 Remdesivir (n = 28)	2020 No Remdesivir (n = 110)	Chi-square / p / φ
Hospital stay <14 days	n = 23 (82.1%)	n = 38 (34.5%)	χ²(1) = 18.7, p < 0.001, φ = 0.34
Hospital stay ≥14 days	n = 5 (17.9%)	n = 72 (65.5%)	
ICU admission	n = 10 (35.7%)	n = 34 (30.9%)	χ²(1) = 0.25, p = 0.62, φ = 0.04
Mechanical ventilation	n = 5 (17.9%)	n = 27 (24.5%)	χ²(1) = 0.46, p = 0.50, φ = 0.06

Hospital stay

**Figure 1:**
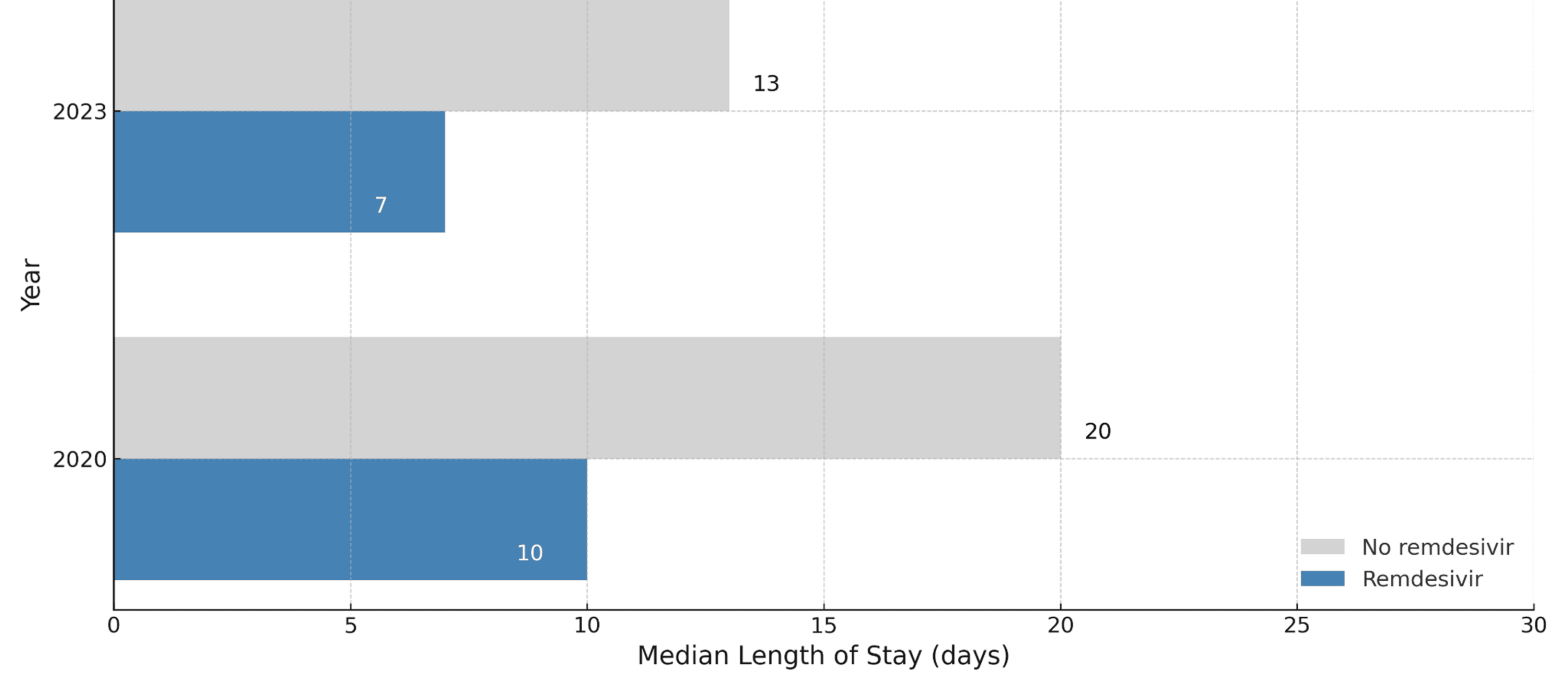
Hospital length of stay among survivors in 2020 and 2023 Bar chart illustrating the median length of hospital stay among survivors treated with or without remdesivir during two time periods: the pre-vaccination era (2020) and the Omicron/post-vaccination era (2023). Patients treated with remdesivir had a shorter median length of stay in both years (10 vs. 20 days in 2020; 7 vs. 13 days in 2023). Gray bars represent patients not treated with remdesivir; blue bars represent those who received remdesivir.

Among survivors, remdesivir was associated with significantly shorter hospital stays in both years (Figure 1):

2020: median 10 days (IQR 8-15) vs. 20 days (IQR 14-31), p = 0.0041; 2023: median 7 days (IQR 6-10) vs. 13 days (IQR 9-17), p = 0.0035

Categorical analysis of hospital stay (<14 vs. ≥14 days) showed significance for both years:

2020: χ²(1) = 8.42, p = 0.004, φ = 0.23; 2023: χ²(1) = 7.91, p = 0.005, φ = 0.21

Mortality

In-hospital mortality was 16.9% (n = 26/154) in 2020 and 9.0% (n = 16/178) in 2023.

2020: Mortality in the remdesivir group was 26.3% (10/38) vs. 13.8% (16/116) in the non-remdesivir group (χ²(1) = 2.75, p = 0.097, φ = 0.13).

2023: Mortality in the remdesivir group was 6.3% (4/64) vs. 10.5% (12/114) in the non-remdesivir group (χ²(1) = 1.37, p = 0.24, φ = 0.09).

Although neither difference reached statistical significance, a trend toward reduced mortality with remdesivir was observed in 2023 (Table 3).

**Table 3:** Hospital length of stay (LOS) by remdesivir use in 2020 and 2023 Abbreviations: LOS, length of stay; IQR, interquartile range; φ, phi coefficient

Variable	2020 Remdesivir (n = 38)	2020 No Remdesivir (n = 116)	2020 Chi-square / p / φ (phi coefficient)	2023 Remdesivir (n = 64)	2023 No Remdesivir (n = 114)	2023 Chi-square / p / φ (phi coefficient)
Hospital stay (median, interquartile range)	10 (8–15)	20 (14–31)		7 (6–10)	13 (9–17)	
Length of stay <14 days	30 (78.9%)	41 (35.3%)	χ²(1) = 8.42, p = 0.004, φ = 0.23	55 (85.9%)	72 (63.2%)	χ²(1) = 7.91, p = 0.005, φ = 0.21
LOS ≥14 days	8 (21.1%)	75 (64.7%)		9 (14.1%)	42 (36.8%)	

ICU admission and mechanical ventilation

ICU admission occurred in 31.2% of patients (48/154) in 2020 and 19.7% (35/178) in 2023. Among ICU patients, in 2020, 66.7% (32/48) required mechanical ventilation, and, in 2023, 42.9% (15/35) required mechanical ventilation.

The differences in ICU admission and mechanical ventilation between remdesivir and non-remdesivir groups were not statistically significant (p > 0.05 in all comparisons), although ICU burden was lower in 2023 overall (Table 4).

**Table 4:** ICU admission and mechanical ventilation Abbreviations: LOS, length of stay; IQR, interquartile range; φ, phi coefficient

Variable	2020	2023	Chi-square / p / φ
ICU admission	n = 48 (31.2%)	n = 35 (19.7%)	χ²(1) = 5.22, p = 0.022, φ = 0.13
Mechanical ventilation (among ICU patients)	n = 32/48 (66.7%)	n = 15/35 (42.9%)	χ²(1) = 4.27, p = 0.039, φ = 0.19

Inflammatory biomarkers

Inflammatory biomarker levels at admission were significantly associated with in-hospital mortality in both cohorts. Non-survivors consistently exhibited higher levels of CRP, LDH, PCT, leukocyte count, and lower lymphocyte percentages.

In 2020, median CRP was 150 mg/L in non-survivors vs. 71 mg/L in survivors (p < 0.001), LDH was 420 U/L vs. 285 U/L (p = 0.003), and PCT was 0.45 ng/mL vs. 0.11 ng/mL (p = 0.006). In 2023, CRP was 139 mg/L vs. 66 mg/L (p < 0.001), LDH 392 U/L vs. 273 U/L (p = 0.005), and PCT 0.39 ng/mL vs. 0.13 ng/mL (p = 0.007) (Tables 5-6).

**Table 5:** Inflammatory biomarkers and mortality (2020) Abbreviations: CRP, C-reactive protein; LDH, lactate dehydrogenase; PCT, procalcitonin; WBC, white blood cell count; IQR, interquartile range; φ, phi coefficient

Variable	Survivors (Median (IQR))	Non-survivors (Median (IQR))	Statistical Test	Chi-square / p / φ (Threshold Category)
CRP (mg/L)	71 (46–107)	150 (110–200)	p < 0.001	χ²(1) = 7.9, p = 0.005, φ = 0.19 (CRP > 100 mg/L)
LDH (U/L)	285 (235–360)	420 (385–540)	p = 0.003	χ²(1) = 6.8, p = 0.009, φ = 0.17 (LDH > 350 U/L)
Procalcitonin (ng/mL)	0.11 (0.06–0.25)	0.45 (0.21–1.10)	p = 0.006	χ²(1) = 5.7, p = 0.017, φ = 0.15 (PCT > 0.5 ng/mL)
Leukocytes (10⁹/L)	7.1 (5.5–9.4)	12.3 (8.0–16.0)	p = 0.008	χ²(1) = 6.5, p = 0.011, φ = 0.16 (WBC > 10 × 10⁹/L)
Lymphocytes (%)	19.8 (14.6–27.4)	8.7 (5.2–13.0)	p < 0.001	χ²(1) = 9.1, p = 0.003, φ = 0.20 (Lymphocytes < 10%)

**Table 6:** Inflammatory biomarkers and mortality (2023) Abbreviations: CRP, C-reactive protein; LDH, lactate dehydrogenase; PCT, procalcitonin; WBC, white blood cell count; IQR, interquartile range; φ, phi coefficient

Variable	Survivors (Median (IQR))	Non-survivors (Median (IQR))	Statistical Test	Chi-square / p / φ (Threshold Category)
CRP (mg/L)	66 (44–99)	139 (95–182)	p < 0.001	χ²(1) = 6.1, p = 0.013, φ = 0.16 (CRP > 100 mg/L)
LDH (U/L)	273 (228–328)	392 (351–475)	p = 0.005	χ²(1) = 5.4, p = 0.020, φ = 0.14 (LDH > 350 U/L)
Procalcitonin (ng/mL)	0.13 (0.07–0.29)	0.39 (0.17–0.89)	p = 0.007	χ²(1) = 4.2, p = 0.041, φ = 0.12 (PCT > 0.5 ng/mL)
Leukocytes (10⁹/L)	6.8 (5.1–9.0)	9.7 (7.1–13.4)	p = 0.010	χ²(1) = 5.0, p = 0.025, φ = 0.13 (WBC > 10 × 10⁹/L)
Lymphocytes (%)	21.2 (15.7–29.1)	9.4 (5.8–14.2)	p < 0.001	χ²(1) = 8.5, p = 0.004, φ = 0.18 (Lymphocytes < 10%)
Variable	Survivors (Median (IQR))	Non-survivors (Median (IQR))	Statistical Test	Chi-square / p / φ (Threshold Category)
CRP (mg/L)	66 (44–99)	139 (95–182)	p < 0.001	χ²(1) = 6.1, p = 0.013, φ = 0.16 (CRP > 100 mg/L)
LDH (U/L)	273 (228–328)	392 (351–475)	p = 0.005	χ²(1) = 5.4, p = 0.020, φ = 0.14 (LDH > 350 U/L)
Procalcitonin (ng/mL)	0.13 (0.07–0.29)	0.39 (0.17–0.89)	p = 0.007	χ²(1) = 4.2, p = 0.041, φ = 0.12 (PCT > 0.5 ng/mL)
Leukocytes (10⁹/L)	6.8 (5.1–9.0)	9.7 (7.1–13.4)	p = 0.010	χ²(1) = 5.0, p = 0.025, φ = 0.13 (WBC > 10 × 10⁹/L)
Lymphocytes (%)	21.2 (15.7–29.1)	9.4 (5.8–14.2)	p < 0.001	χ²(1) = 8.5, p = 0.004, φ = 0.18 (Lymphocytes < 10%)

Categorical analyses confirmed significant associations between elevated biomarker thresholds and mortality. In both years, CRP > 100 mg/L, LDH > 350 U/L, and PCT > 0.5 ng/mL were significantly more frequent among non-survivors (all p < 0.05). Lymphopenia (< 10%) and leukocytosis (> 10 × 10⁹/L) also showed strong correlations with in-hospital mortality.

## Discussion

This retrospective cohort study reinforces previous evidence that remdesivir use is associated with shorter hospital stays among hospitalized COVID-19 patients, both in the pre-vaccination (2020) and post-vaccination (Omicron-dominant, 2023) periods [1-3]. Our findings align with randomized controlled trials and real-world analyses demonstrating that early antiviral intervention can positively influence clinical trajectories, especially in patients requiring oxygen support [4,5].

Sex- and age-related differences persisted in both cohorts, with male patients and older individuals showing higher mortality risk, in line with earlier studies linking these variables to immunological vulnerability and disease severity [6,7]. Additionally, elevated inflammatory markers at admission - including CRP, LDH, procalcitonin, and leukocyte counts - were significantly associated with in-hospital mortality, mirroring earlier findings from Chinese, European, and US cohorts [8-10].

Of particular note, our study identified a consistent relationship between remdesivir treatment and a reduced hospital length of stay among survivors in both years. This was observed despite evolving viral variants, changing vaccination status, and updated treatment protocols. Although in-hospital mortality was not significantly lower in the remdesivir group, a favorable trend emerged in the 2023 cohort, suggesting a potential synergistic effect between remdesivir, early intervention, and improved supportive care [11-13].

Our findings also align with recent Omicron-era studies by Mozaffari et al. [11] and Kuritzkes [12], who reported sustained clinical benefits of remdesivir in high-risk and immunocompromised populations [14,15]. Biomarker-based risk stratification remains essential for therapeutic decisions; early identification of elevated CRP, LDH, or low lymphocyte counts may guide timely antiviral administration [16-18].

Consistent with previous Cochrane reviews and real-world analyses from the US Veterans Health Administration and multicenter trials across North America and Europe, our data support the role of remdesivir in reducing hospitalization burden [19-24]. Notably, although the WHO Solidarity Trial questioned mortality benefits across broader populations [25], multiple national and multicenter cohort studies have affirmed the clinical utility of remdesivir in hospitalized patients with moderate to severe disease [26-29].

Our study further underscores the importance of biomarker-guided decision-making. Early identification of disease severity using biomarkers such as CRP and LDH may help optimize the timing of antiviral treatment and improve outcomes, particularly during high-transmission periods such as the Omicron wave [30].

Limitations

This study has several limitations. First, it is a retrospective, single-center study, which limits generalizability. Second, selection bias may be present, as the decision to administer remdesivir was made by treating physicians based on national guidelines and clinical judgment rather than randomization. Third, the two cohorts (2020 vs. 2023) differed in multiple systemic factors, including vaccination rates, circulating variants, and hospital protocols, which may have influenced outcomes. Fourth, some confounders - such as BMI, smoking status, and detailed comorbidity burden - were not fully captured or adjusted for in multivariate analyses. Finally, the study did not include ICU severity scores (e.g., Sequential Organ Failure Assessment (SOFA) or Acute Physiology and Chronic Health Evaluation II (APACHE II)), which limits insight into critical illness at baseline.

Despite these limitations, the study provides valuable real-world data from two distinct pandemic phases and highlights remdesivir’s potential role in optimizing inpatient care during periods of high clinical burden.

## Conclusions

This real-world cohort study demonstrates that remdesivir treatment was consistently associated with shorter hospital stays among survivors with COVID-19 pneumonia during both the pre-vaccination era (2020) and the Omicron-dominant, post-vaccination period (2023). Although a statistically significant reduction in mortality was not observed, a favorable trend in the 2023 cohort suggests potential benefits linked to earlier treatment, improved clinical protocols, and widespread vaccination.

These findings support the continued use of remdesivir as part of inpatient treatment strategies aimed at alleviating hospital burden and improving patient flow. Further prospective, multicenter studies are warranted to better define its effectiveness across diverse patient populations, disease severities, and viral variants.
